# Bone resorption: an actor of dental and periodontal development?

**DOI:** 10.3389/fphys.2015.00319

**Published:** 2015-11-05

**Authors:** Andrea Gama, Benjamin Navet, Jorge William Vargas, Beatriz Castaneda, Frédéric Lézot

**Affiliations:** ^1^Institut National de la Santé et de la Recherche Médicale, UMR-1138, Equipe 5, Centre de Recherche des CordeliersParis, France; ^2^Odontologic Center of District Federal Military PoliceBrasilia, Brazil; ^3^Institut National de la Santé et de la Recherche Médicale, UMR-957, Equipe Ligue Nationale Contre le CancerNantes, France; ^4^Laboratoire de Physiopathologie de la Résorption Osseuse et Thérapie des Tumeurs Osseuses Primitives, Faculté de Médecine, Université de NantesNantes, France; ^5^Department of Basic Studies, Faculty of Odontology, University of AntioquiaMedellin, Colombia

**Keywords:** bone resorption, RANKL, Zoledronic acid, tooth

## Abstract

Dental and periodontal tissue development is a complex process involving various cell-types. A finely orchestrated network of communications between these cells is implicated. During early development, communications between cells from the oral epithelium and the underlying mesenchyme govern the dental morphogenesis with successive bud, cap and bell stages. Later, interactions between epithelial and mesenchymal cells occur during dental root elongation. Root elongation and tooth eruption require resorption of surrounding alveolar bone to occur. For years, it was postulated that signaling molecules secreted by dental and periodontal cells control bone resorbing osteoclast precursor recruitment and differentiation. Reverse signaling originating from bone cells (osteoclasts and osteoblasts) toward dental cells was not suspected. Dental defects reported in osteopetrosis were associated with mechanical stress secondary to defective bone resorption. In the last decade, consequences of bone resorption over-activation on dental and periodontal tissue formation have been analyzed with transgenic animals (*RANK*^*Tg*^ and *Opg*^−∕−^ mice). Results suggest the existence of signals originating from osteoclasts toward dental and periodontal cells. Meanwhile, experiments consisting in transitory inhibition of bone resorption during root elongation, achieved with bone resorption inhibitors having different mechanisms of action (bisphosphonates and RANKL blocking antibodies), have evidenced dental and periodontal defects that support the presence of signals originating bone cells toward dental cells. The aim of the present manuscript is to present the data we have collected in the last years that support the hypothesis of a role of bone resorption in dental and periodontal development.

## Introduction

**Figure 1:**
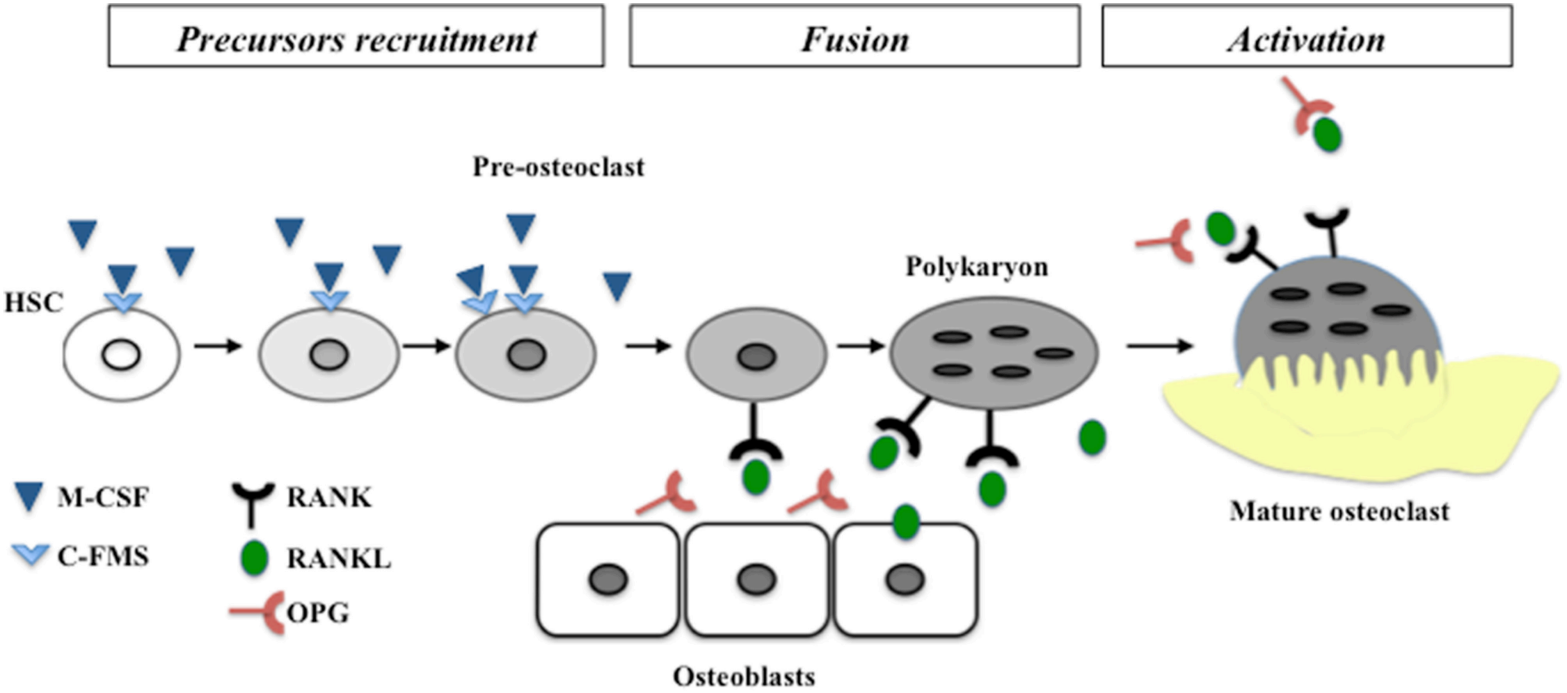
**Schematic representation of different steps of osteoclastogenesis**. The two mains factors controlling the differentiation called M-CSF and RANKL are represented with their receptors. M-CSF is necessary to recruitment and expression of RANK at the surface of pre-osteoclasts. RANKL enable the fusion of pre-osteoclasts in polykaryon and the final differentiation in mature osteoclasts. HSC, hematopoietic stem cell.

Early tooth development, more precisely initiation and morphogenesis, has been extensively studied in the last decades. Factors implicated in the cross-talk between epithelial and mesenchymal cells have been identified (for review Mitsiadis and Graf, 2009). Regarding later stages of tooth development, more precisely dental and periodontal histogenesis, the differentiation processes of mineralized tissue forming cells (namely amelogenesis, dentinogenesis and cementogenesis) have also been widely studied (Foster et al., 2007; Babajko et al., 2014; Bleicher, 2014). Pathologies associated with dysfunctions of these processes are nowadays well characterized as amelogenesis imperfecta and dentinogenesis imperfecta (Cobourne and Sharpe, 2013). Dental and periodontal histogenesis corresponds to an important volumetric growth of these tissues, more precisely regarding root formation. Consequently, the surrounding alveolar bone has to be remodeled simultaneously to enable normal tooth development and a dental functional achievement through the eruption process. Bone remodeling requires differentiation and activity of bone resorbing cells from hematopoietic origin called osteoclasts. Osteoclastogenesis is a well-characterized process with three consecutive steps corresponding to precursors recruitment, their fusion into mature polynucleated osteoclasts and finally the activation of these mature osteoclasts (Lézot et al., 2010). The two major signaling factors implicated in these differentiation steps are presented in Figure 1.

**Figure 2:**
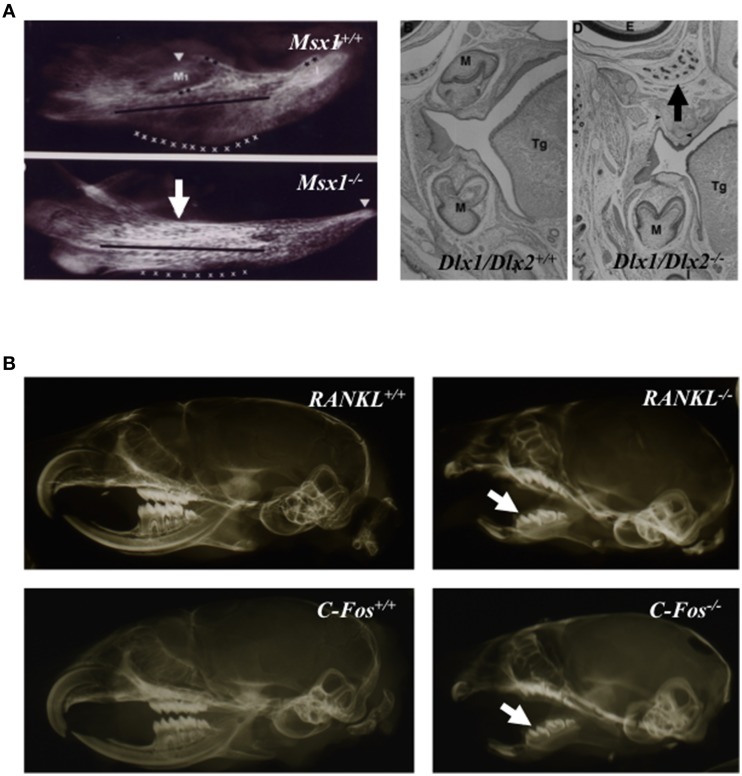
**Tooth agenesis consequence on alveolar bone formation (A) and close to normal crown morphology in osteopetrotic animals (B)**. Mandible micro-radiographies and head frontal sections of newborn *Msx1* and *Dlx1/Dlx2* null mutants **(A)**; Msx1 null mutant mouse image taken form Orestes-Cardoso et al. (2002). Absence of alveolar bone formation in area of tooth agenesis (arrows). Micro-radiographies of wild type, *C-Fos*^−∕−^ and *RANKl*^−∕−^ 21 day-old mouse heads **(B)** the formation of dental crowns (arrows) in osteopetrotic mutant mice with morphologies close to those of control littermate mice. M, molar; Tg, tongue.

Signals coming from dental and periodontal tissues were shown to stimulate the alveolar bone remodeling (Wise, 2009). Indeed, these tissues secrete factors stimulating osteoclastogenesis (Wise, 2009). The absence of alveolar bone formation in the case of dental agenesis (Figure 2A) supported the assertion that dental and periodontal tissues are central coordinator elements in the development of the dento-alveolar bone complex. Moreover, the apparently normal development of the crown region observed in osteopetrotic mouse models (Figure 2B), despite the altered bone resorption, suggests that crown mineralized tissue formation is independent of bone resorption.

In this context it was not surprising that potential reverse signals from bone cells toward dental and periodontal tissues have been rarely considered. Recently, the analysis of dental and periodontal development in mouse models of hyper-resorption (*RANK*^*Tg*^ and *Opg*^−∕−^) has changed the vision of the dento-alveolar bone complex development (Castaneda et al., 2011, 2013). Bone resorption was for the first time shown to be an active element of the dental and periodontal tissues development. This active implication was supported by results of studies comprising transitory inhibition of bone resorption during dental and periodontal tissue histogenesis, achieved with bisphosphonate or RANKL blocking antibody (Lézot et al., 2014, 2015). In these studies dental and periodontal tissue defects were associated and proportional to the induced delay of tooth eruption.

Here we present a hypothetical model of tooth root and periodontal development based on our own results as well as on previously reported by other findings.

## Current status concerning tooth root and periodontal formation: Facts and hypotheses

### Consequences of RANK over-expression in the monocyte-macrophage lineage (*RANK*^*Tg*^ mouse) on dental and periodontal development

**Figure 3:**
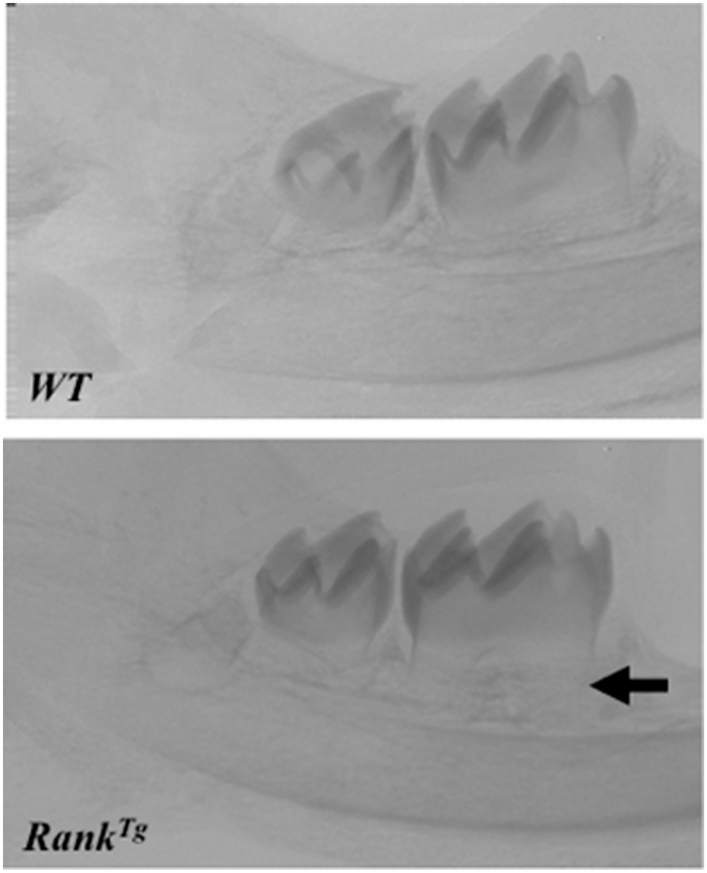
**Accelerated root elongation in ***RANK***^***Tg***^ mouse**. Micro-CT section in the mandible main axis show in 11 day-old *RANK*^*Tg*^ mouse a more advanced root elongation (arrow) compared to wild type mouse.

**Figure 4:**
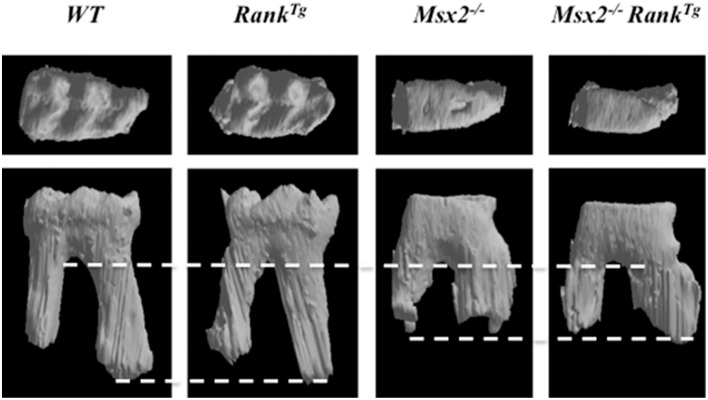
**Roots and crown morphologies of the mandible first molars of adult wild-type, ***RANK***^***Tg***^, ***Msx2***^−∕−^, and ***Msx2***^−∕−^***RANK***^***Tg***^mice**. Roots of *RANK*^*Tg*^ mouse molars are thinner and roots of *Msx2*^−∕−^ mouse molars are shorter as evidenced by dash lines. Crown of *Msx2*^−∕−^^−∕−^ mouse are flat with no cuspide independently of RANK over-expression.

In order to analyze the consequences of RANK over-expression on dental and periodontal tissue growth, a transgenic mouse-line overexpressing *RANK* in the osteoclast precursors (*RANK*^*Tg*^; Duheron et al., 2011) was used. The dental and periodontal phenotype of *RANK*^*Tg*^ mouse was analyzed comparatively to littermate from birth to 1 month (Castaneda et al., 2011). Results show a significant increase in the osteoclast number around the tooth at all ages. This led to an earlier tooth eruption and an accelerated tooth root elongation (Figure 3). The final root length is not affected (Figure 4) but an important reduction of the root diameter is observed no matter what the genetic background (wild-type or *Msx2* null mutant) considered (Castaneda et al., 2011, 2013).

Interestingly, the complex phenotype of *Msx2*^−∕−^ mouse combining amelogenesis imperfecta, root dysmorphia (defects in Hertwig epithelial root sheaths (HERS) and epithelial rests of Malassez), mild-osteopetrosis (with *RANKl* expression severely decreased in the dental epithelium and alveolar bone), and dentinogenesis imperfecta (Aïoub et al., 2007; Molla et al., 2010; Berdal et al., 2011) was partly rescued by RANK over-expression (Castaneda et al., 2013). Indeed, RANK over-expression resulted in significant recovery of all molar eruption and root elongation processes (Figure 4). However, the roots remained shorter than in wild-type mice and no improvement of the crown morphology was observed (Figure 4).

These results show that root length is genetically determined while root thickness is environmentally controlled, specifically by the bone resorption ability.

**Figure 5:**
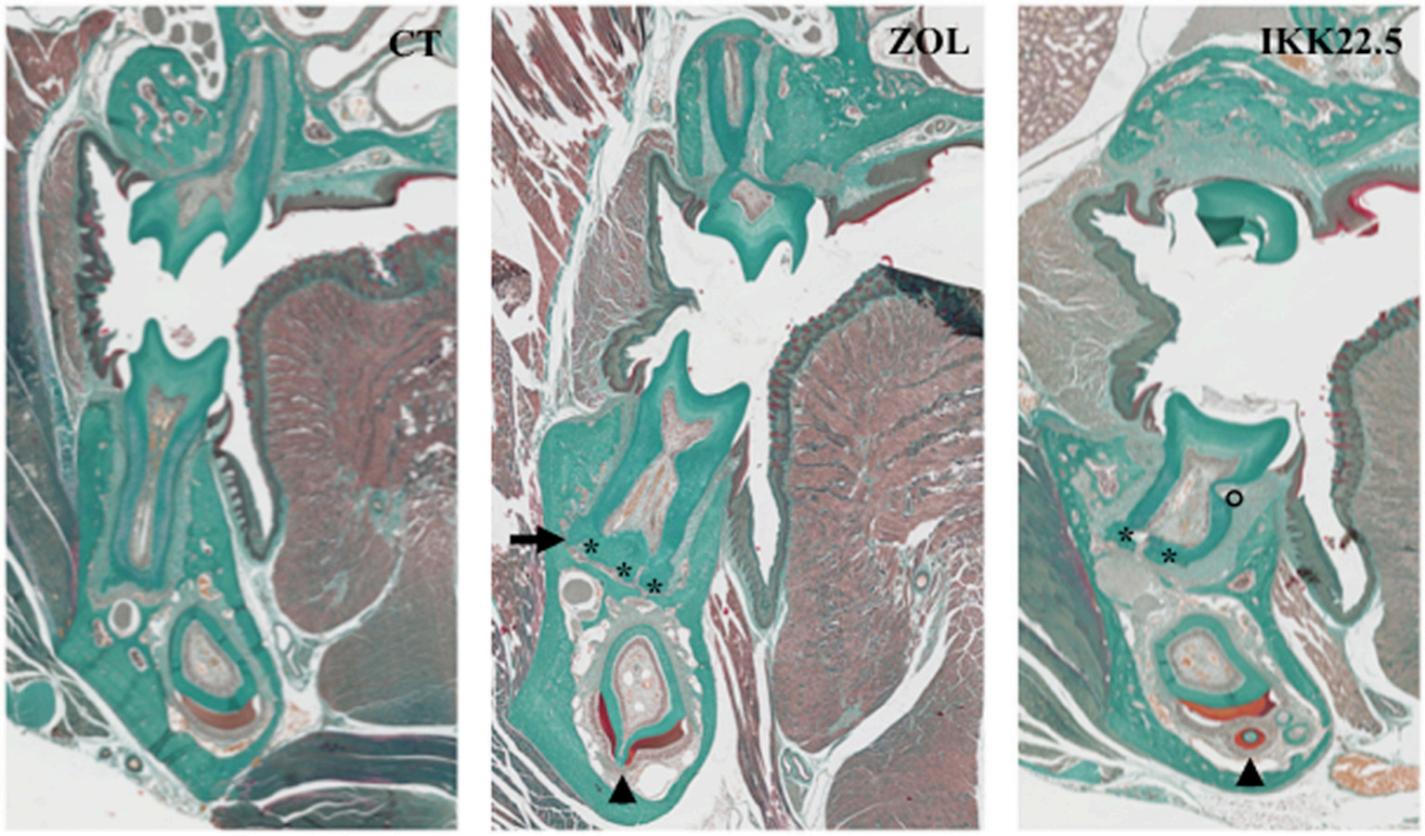
**Dental and periodontal consequences of four injections of ZOL or IKK22.5 in newborn mice, 3 months after the last injection**. Head frontal sections in the plane of the first molars show the presence of ankylosis (arrow), hyper-cementosis (stars), enamel organ disorganization (arrow-heads), and root resorption (circle) in treated animals.

The complete analysis of the *RANK*^*Tg*^ mouse dento-alveolar bone complex phenotype has so enabled to demonstrate that bone resorption is an important element of dental and periodontal tissue development (Castaneda et al., 2011, 2013). RANK over-expression induces an early tooth eruption and root elongation with, as a final consequence, a reduction of the root diameter. This accelerated tooth root elongation corresponds to an increase of HERS cells and adjacent follicular sac mesenchyme cells proliferation (Castaneda et al., 2011). The final root lengths of the RANK transgenic and wild type mice are similar suggesting that the interactions between epithelial and mesenchyme cells are correct but accelerated (Castaneda et al., 2011). The *Msx2*^−∕−^ mouse present many defects of the root formation (Aïoub et al., 2007) including an important reduction of the length as shown in Figure 5. The fact that, in the *Msx2*^−∕−^ mouse the RANK over-activation has no repercussion on the final root length validates that root length is genetically determined. Meanwhile, the root diameter appears to be micro-environmentally controlled, more specifically by the bone resorption capability (Castaneda et al., 2011, 2013). Finally the reverse relationship between bone resorption level and the root diameter established by these studies (Castaneda et al., 2011) could explain part of the root defects seen in diseases with perturbations of the osteoclast function. Concerning the root resorption observed in these diseases, the question of a relationship between root thinness and the prevalence of root resorption is raised. Interestingly, such thin root resorptions are observed in the *Opg*^−∕−^ mouse (Koide et al., 2013) in the context of a progressive loss of the alveolar bone.

In humans, *RANK* gene gains of function mutations have been found in three seemingly distinctive disorders (the Familial Expansile Osteolysis, the Expansile Skeletal Hyperphosphatasia and the Early-onset Paget Disease of Bone). These mutations increase the RANK signal peptide length and alter its normal cleavage, what is believed to cause a NF-κB pathway over-activation (Whyte and Hughes, 2002; Nakatsuka et al., 2003). Such over-activation of the RANK-signaling pathway causes a hyper-osteoclastic activity that increases the bone turnover. A notable observation in these patients is an early tooth loss associated in some case with an idiopathic external resorption localized at either apical or cervical levels (Mitchell et al., 1990; Hughes et al., 1994; Whyte, 2006). This convergence of phenotype between human patients and *RANK*^*Tg*^ mice qualified the *RANK*^*Tg*^ mouse as a model of these three different pathologies and confirmed the importance of bone resorption for dental and periodontal tissue development.

### Consequences of transitory inhibition of bone resorption using zoledronic acid or a RANKL blocking antibody on dental and periodontal development

In order to analyze the consequences of transitory inhibitions of bone resorption on dental and periodontal tissue growth, a powerful pharmacologic inhibitor of bone resorption from the bisphosphonate family was injected (four injections in total every 2 days) in newborn or 1 week-old mice. The impact on dental and periodontal tissues was analyzed at the end of treatment, 1 and 3 months after the last injection. Zoledronic acid (ZOL), a third generation bisphosphonate, was chosen for experiments and C57BL/6J and CD1 mice used.

The different molars were not similarly affected by the treatment. A relationship appears between severity of dental and periodontal defects and each molar developmental period encompassed by the treatment. Indeed, when injections were performed in newborn pups, the first molar was the most affected and the third molar the least affected (Lézot et al., 2014, 2015). The C57BL/6J mice appear to be more sensitive to ZOL than the CD1 mice who need more elevated doses to obtain similar effects (Lézot et al., 2015). In addition to delayed eruption, the main observed defects of dental and periodontal tissues were abnormal amelogenesis with disorganized ameloblasts, root ankylosis, hypercementosis and with time presence of root resorption (Figure 5; Lézot et al., 2014). These results evidence that transitory inhibition of bone resorption with ZOL irreversibly impact the histogenesis of dental and periodontal tissues with long-term consequences that remain to be evaluated.

Similar experiments were performed with another powerful inhibitor of bone resorption, a RANKL blocking antibody named IKK22.5.

Experimental results evidenced that while C57BL/6J mice had several teeth included, CD1 mouse had only the upper first molars included confirming the difference of sensitivity to bone resorption inhibitors between these two mouse strains (Lézot et al., 2015). Regarding the dental and periodontal phenotype of non-included molars of CD1 mouse, similar defects to those induced by ZOL injections were observed (Figure 5). Interestingly, after the end of treatment with the IK22.5 antibody, a shorter period is necessary to observe neo-osteoclasts presence on the alveolar bone surface than observed after ZOL treatment (Lézot et al., 2015) signaling a more stable inhibition with ZOL than with IKK22.5.

These results demonstrate that transitory use of two different pharmacological bone resorption inhibitors during root elongation induces dental and periodontal defects. This supports our hypothesis of the existence of signaling from bone cells toward dental cells. These powerful pharmacological inhibitors were developed for the treatment of pathologies characterized by excessive bone resorption such as juvenile Paget's disease, osteoporosis, primary or metastatic bone tumors and familial expansile osteolysis (Silverman, 2011; Zwolak and Dudek, 2013; Tella and Gallagher, 2014). In pediatric patients, a RANKL-blocking antibody (Denosumab) is currently under clinical evaluation for osteogenesis imperfecta (phase 2 clinical trial NCT01799798) and for Giant Cell Tumor of Bone (phase 2 clinical trial NCT00680992) with promising preliminary reports in both cases (Semler et al., 2012; Chawla et al., 2013; Karras et al., 2013; Demirsoy et al., 2014; Federman et al., 2014). Bisphosphonates are currently used for the treatment of osteogenesis imperfecta (Barros et al., 2012; Bishop et al., 2013; Ward and Rauch, 2013; Sousa et al., 2014) and juvenile Paget's disease (Demir et al., 2000; Cundy et al., 2004; Polyzos et al., 2010; Saki et al., 2013). In addition, they are under evaluation for treatment of primary bone tumors (Goldsby et al., 2013; phase 3 Clinical trials NCT00987636, NCT00742924, and NCT004470223) and Fibrous Dysplasia of Bone (phase 2 clinical trial NCT00445575). Concerning all these young patients, dental and periodontal tissue developmental defects may occur as a consequence of the bone resorption inhibition. Preclinical studies and clinical observations have already demonstrated that bisphosphonates, in particular alendronate and ZOL, delay or inhibit tooth eruption causing several dental abnormalities (Grier and Wise, 1998; Bradaschia-Correa et al., 2007; Kamoun-Goldrat et al., 2008; Hiraga et al., 2010) and may, as in the juvenile Paget's disease of bone, exert an inhibitory effect on bone mineralization (Polyzos et al., 2011). Such inhibition of mineralization has been observed for various bisphosphonate in *in vitro* tests using calvaria osteoblast culture (Widler et al., 2002).

## Conclusion

The data presented in this review are unambiguous concerning the role of bone resorption on the development of dental and periodontal tissues and supports the hypothesis of a direct implication of osteoclasts in dental and periodontal tissue formation. Further studies will be necessary to decipher, at the molecular level, signals originating from bone cells (presumably osteoclasts) toward dental and periodontal cells. These signals could be directly secreted by bone cells or released from the bone matrix during the resorption.

## Funding

The presented projects have received the financial support of the French Association for Cancer Research (ARC, Project # ECL;2010R00778), the “ligue contre le cancer” Association, the Liddy Shriver Sarcoma Initiative (grant) and the French National Cancer Institute (Funding INCa-6001).

### Conflict of interest statement

The authors declare that the research was conducted in the absence of any commercial or financial relationships that could be construed as a potential conflict of interest.
